# NIH’s 15% cap: a cost comparison and research outlook

**DOI:** 10.1038/s44319-025-00418-4

**Published:** 2025-03-18

**Authors:** Shina Caroline Lynn Kamerlin, Mikael H Elias

**Affiliations:** 1 School of Chemistry and Biochemistry, Georgia Institute of Technology, 901 Atlantic Drive NW, Atlanta, GA 30332 USA; 2 Department of Biochemistry, Molecular Biology and Biophysics, University of Minnesota, St. Paul, MN 55108 USA; 3 Biotechnology Institute, University of Minnesota, St. Paul, MN 55108 USA

**Keywords:** Careers, Economics, Law & Politics, Science Policy & Publishing

## Abstract

The decision by the US government to cap the NIH grant overhead rate to 15% will likely underfund the country’s research institutions for their infrastructure relative to their international peers and the private sector. This move could jeopardize the country’s lead in the biological sciences.

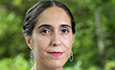

Overnight, leading research institutions in the USA have potentially lost billions of dollars in indirect funding, in a move that will likely change the biomedical research landscape. The recent decision by the US government to cap overhead rates for all research grants from the National Institute of Health (NIH) at 15% has created an extinction-level financial crisis for Research-intensive (R1) universities, where these overheads range between 30 to 70% depending on the university, with an average around 50%. At the time of writing, the execution of this cap is blocked by a restraining order by a federal judge.

Also known as Facilities and Administrative (F&A) costs, grant overheads support essential infrastructure such as building and instrument maintenance, administration, utilities, facilities, equipment, or rent—all items that, directly or indirectly, support cutting-edge research. Historically, these rates were negotiated between a research institution and the US Department of Health and Human Services (HHS) or another federal agency and varied as a function of the institution’s investment into research infrastructure. Within this F&A rate, the administrative portion is capped at 26% and covers accounting, compliance and grant handling, among other things. The portion for facilities costs, such as building, operation, or depreciation, is variable and the main reason for the differences between institutional F&A rates. HHS agreements are 4-years terms and based on negotiations: for example, the F&A rate at the University of Minnesota dropped from 55% to 54% in the latest agreement.

The implications of this decision—a drastic reduction of the current 27–28% average NIH overhead rate to all institutions including non-R1 over all types of grants—are nothing but dire: an annual loss of US$4B in indirect costs to research institutions, including some of the best universities in the USA and globally. At the University of Minnesota alone, the cap would represent a loss of US$100 to US$130 million. Universities in Texas, New York, and California are facing losses that may amount to US$310 million, US$632 million, and US$804 million, respectively. These drastic cuts, if confirmed, will profoundly change the biomedical research landscape. We will likely witness large-scale layoffs, including support staff and research scientists—who are often paid out of indirect costs—as entire research facilities may close down. Universities may have to share research facilities and infrastructures with other regional institutions. Inevitably, whatever the measures, the cuts will reduce the availability of research infrastructure and the research footprint by leading US universities and scientists may be leaving academia and/or the USA due to reduced prospects.

One argument given to justify this reduction is that US institutions are outliers in terms of overhead costs. However, we find that the available data, although imperfect, does not support this notion. In France, where universities and institutions are traditionally provided with block funding from the state to cover their base expenses, recent reforms of the Agence Nationale de la Recherche (ANR) introduced a composite overhead rate (Préciput) that approximates about 30% of a project’s funding. The UK Research and Innovation (UKRI) funds 80% of the Full Economic Cost of a given project. In Germany, the Deutsche Forschungsgemeinschaft (DFG) provides an overhead of 22% to cover additional indirect costs, and the Swiss National Science Foundation (SNF) gives 20%. We were unable to locate data for China or India. Hence, the historical NIH rate with an average of 27–28% for all institutions and all grant types is coherent with other major international funding agencies. Worse, a 15% cap would make US research institutions significantly underfunded compared to their counterparts elsewhere.

Another way to evaluate the argument for reducing NIH overheads is to benchmark them with industry standards. The equivalent of F&A in the biomedical industry is not easily accessible. However, the Selling, General and Administrative (SG&A) expenses represent a proxy for overheads: these account for all expenses that are not directly tied to the production of goods or services, such as administrative costs, facilities, and support. While not a perfect equivalent to university F&A rates due to different calculation basis and expenses, SG&A-to-revenue ratios can be useful for evaluating overhead burdens across these different sectors.

Data for biotechnology companies show that overhead rates are similar to NIH’s traditional rates. For example, in 2022, the average SG&A-to-sales ratio among 15 leading pharmaceutical firms was 40.5% compared to 39.6% in 2021. The same year, AstraZeneca, Novartis, and Johnson & Johnson had 51.9%, 46.7%, and 43.6% SG&A-to-revenue ratios, respectively. The average SG&A-to-revenue ratios for a sample of small and mid-cap drug companies is 34%. Outside the USA, Korean publicly traded biotech companies show a 30% SG&A rate (Lee, 2021). Remarkably, smaller biotech companies tend to have even higher SG&A rates, perhaps accounting for their extensive investment in research infrastructure and equipment.

Evidently, these numbers show that both academic and private-sector research in biotechnology require substantial indirect investments. Interestingly, the median SG&A-to-revenue ratio for large pharmaceutical companies (28.2%; Ledley et al, 2020) is very close to the historic NIH rate for academic institutions (27–28%). In contrast, non-pharma S&P 500 companies report a much lower median SG&A-to-revenue ratio (16.6%; Ledley et al, 2020), strengthening the notion that biotechnology research inherently depends on larger overheads. We are aware that the comparisons are imperfect. Yet, the observation that biomedical research, including translational research, require uniquely high overhead rates sticks out. This is possibly due to its requirement for cutting-edge research facilities and equipment and their constant upgrade to keep up with the rapid pace of technological progress.

Another justification for reducing the F&A rate is that indirect costs may not contribute directly to research. However, this viewpoint misses the importance of research facilities, equipment maintenance, infrastructure, compliance and administrative support to create new knowledge and improve health: NIH funding contributed to all 210 new drugs approved by the US Food and Drug Administration between 2010 and 2016 (Galkina Cleary et al, 2018).

It is unclear whether this cap will extend to other federal funders, such as the National Science Foundation, the Department of Energy, the Department of Agriculture or the Department of Defense that also fund a large portion of biological sciences. While some of these funders operate with a different model and include indirect costs in the total budget, these agencies also contribute significantly to university research infrastructures. If extended, the consequences could be even more damaging.

Concurrently, the USA is witnessing layoffs at research agencies; as of the time of writing, NIH recently removed 1200 employees, including admin staff, scientists and tenure-track investigators, and more job cuts could occur (Kaiser, 2025a) as well as the non-renewal of senior scientists (Kaiser, 2025b). Layoffs also occurred at NSF and the US Centers for Disease Control and Prevention among other agencies. New grants and renewals are awarded at very slow pace, study sections are frozen which creates grant application review and processing backlog (Kozlov, 2025) for agencies that already need to adjust to their reduced workforce. Research labs across the USA that depend on NIH research awards are bracing for the reduction of their rosters as funding is largely paused, which will result in much reduced research capacity and ultimately, output. Already, numerous graduate schools have paused or drastically reduced graduate student recruitment, which will undermine the training of the next generation of scientists. In parallel, hiring freezes and budget cuts have already started.

As we write these lines, the US Senate voted on a resolution that would increase the overall general science, space and technology budget over the next 10 years. Also recently, the continuing House GOP resolution narrowly passed on February 26th 2025, and included a commitment to reduce spending by at least US$1.5 trillion over the next 10 years, and therefore with a possibility of reduced science funding as part of this effort. In the positive scenario—or even at a constant budget—this could mean that more projects would be funded. Yet, if the overhead cap is upheld, it is unclear what the overall outcome will be, with more funded projects and underfunded critical infrastructure to support them. Overall, this is adding to the general uncertainty of the scientific community in the USA and endangers the country’s leadership in the biotechnology sector.

While concerns about efficiency are always justified and even sometimes needed, capping NIH overhead at 15% is unlikely to make universities more efficient. The likely output is not a more streamlined research ecosystem, but rather the reduction of research facilities, specialized labs and a large-scale, long-term decline in research capabilities that may take decades to recover from. Reform can be a great thing, and both higher-education institutions and the research system could certainly be transformed to increase their respective efficiency and output. Yet, a nuanced approach, balancing reforms with the specific needs of biomedical research for costly infrastructures and the imperative of scientific progress for economic growth, is needed.

## Supplementary information

Peer Review File

## References

[CR1] Galkina Cleary E, Beierlein JM, Khanuja NS, McNamee LM, Ledley FD (2018) Contribution of NIH funding to new drug approvals 2010–2016. Proc Natl Acad Sci USA 115:2329–2334 29440428 10.1073/pnas.1715368115 PMC5878010

[CR2] Kaiser J (2025a) More NIH job cuts coming? Agency’s scientists already reeling after week of firings. Science. 10.1126/science.z9jsxp1

[CR3] Kaiser J (2025b) NIH ban on renewing senior scientists adds to assaults on its in-house research. Science. 10.1126/science.zuhita3 10.1126/science.adx2122 40048536

[CR4] Kozlov M (2025) Revealed: NIH research grants still frozen despite lawsuits challenging Trump order. Nature 638:870–871 39979573 10.1038/d41586-025-00540-2

[CR5] Ledley FD, McCoy SS, Vaughan G, Cleary EG (2020) Profitability of large pharmaceutical companies compared with other large public companies. JAMA 323:834–843 32125401 10.1001/jama.2020.0442 PMC7054843

[CR6] Lee N (2021) The association between the selling, general & administrative expenses and age at IPO of biotech companies. Acad Account Financ Stud J 25:1–11

