# Supplementary material for: NIH’s 15% cap: a cost comparison and research outlook
Source: EMBO Rep. 2025 Mar 18;26(7):1676–8. doi: 10.1038/s44319-025-00418-4 (PMC11977179; doi:10.1038/s44319-025-00418-4)
Supplement: Supplementary file 1 — Peer Review File [file 44319_2025_418_MOESM1_ESM.pdf]

EMBO reports

NIH’s 15% Cap: A cost comparison and research outlook

Shina Caroline Lynn Kamerlin and Mikael Elias

Corresponding author(s): Mikael Elias (mhelias@umn.edu)

Review Timeline:

Submission Date:	27th Feb 25
Accepted:	4th Mar 25

Editor: Holger Breithaupt

Transaction Report: This article type is not peer reviewed.
